# Supplementary material for: Abundance of Microvascular Endothelial Cells Is Associated with Response to Chemotherapy and Prognosis in Colorectal Cancer
Source: Cancers (Basel). 2021 Mar 23;13(6):1477. doi: 10.3390/cancers13061477 (PMC8004880; doi:10.3390/cancers13061477)
Supplement: Supplementary file 1 [file cancers-13-01477-s001.pdf]

SUPPLEMENTAL MATERIALS

Table S1. Member genes of the microvascular endothelial (mvE) cells score.

ABCB9	ABCD4	ABCF2	ACE	ACTG1	ACTL7A	ACTR1A	ACVRL1	ADRA1B	AHDC1
AKAP4	ANGPT2	ANO2	ANXA2	AP2M1	ARF1	ARHGEF15	ARL2BP	ARPC1A	ART4
ARVCF	ATF6	ATF7	ATP1B3	ATP2B3	ATP6V0E1	ATXN2L	AURKAIP1	B3GALT5	BANF1
BCL10	BFAR	BMX	BYSL	C16orf62	C19orf24	C1orf123	CALM1	CAPN11	CASP10
CAV1	CAV2	CCDC90B	CCKAR	CCNG1	CD34	CD9	CD93	CDC27	CDC37
CDK9	CEACAM21	CECR5	CETP	CHCHD2	CHST12	CIAPIN1	CISD1	CLDN14	CLDN5
CLEC1A	CLEC4M	CLIC1	CLPP	CLTA	CNIH4	COMMD4	COPA	COPS6	COX4I1
COX7A2L	CPA1	CPSF3L	CSF2RB	CSNK2A2	CTNNA1	CXorf36	DAD1	DCAF7	DCTN2
DCTN5	DCTPP1	DDA1	DDX56	DEF8	DHX38	DLST	DNAJC7	DNPEP	DYNC1H1
DYNC1LI2	DYRK1B	ECD	ECHS1	EDC3	EDC4	EI24	EIF2B2	EIF3K	EIF4E2
EIF4G1	ELK4	ELOVL1	EMCN	ENOPH1	ENTPD1	ERG	ERH	EXD2	EXOC7
F11R	FAM107A	FAM124B	FAM65A	FAM96B	FBXO22	FDPS	FLOT2	FLT1	FLT4
FN3K	FNDC8	FOXC2	FOXD3	FRMD8	FRS3	FTSJ3	GABPB1	GANAB	GDI2
GEMIN7	GFOD2	GIMAP4	GIMAP6	GIPC1	GIT1	GJA4	GLRX3	GMPR2	GOLGA3
GOT2	GPKOW	GPN2	GPR4	GSDMD	GTF3C5	GYG1	HADHA	HCFC1	HCRTR1
HDAC1	HERC1	HOXD3	HSP90AB1	HSPA4	HSPB11	HTATIP2	HTR1B	HYAL2	IL3RA
ILF2	INPP5E	INPP5K	ITGA9	KANK3	KAT5	KCNAB1	KCTD2	KDR	KEAP1
KIF17	LAMP1	LGALS1	LMBR1L	LRRC3	LRRFIP1	LSG1	LYL1	LYPLA1	LYPLA2
LYVE1	LYZL6	MAGEB1	MAP3K3	MAPK12	MAPK3	MAT2B	MBTPS1	MED16	MED20
METTL3	MGAT5	MIF	MIP	MMRN1	MMRN2	MMS19	MOSPD3	MRPL15	MRPL17
MRPL9	MRPS16	MRPS28	MTCH1	MTCH2	MTG1	MTHFR	MTMR2	MUL1	MYCT1
MYL12A	MYL6	N4BP3	NAP1L4	NCBP2	NCK1	NDUFB11	NDUFC2	NECAP2	NEDD8
NOC2L	NOTCH4	NOVA2	NPR1	NUBP2	NUP188	OXA1L	P4HB	PABPC4	PCDH12
PCDHA6	PCGF3	PDCL	PGLS	PIAS4	PIK3CG	PITPNB	PLCG1	PLD2	PLS3
PLVAP	PLXNB3	PMPCA	PNP	POLR2F	POLR2J	POM121L2	PPM1F	PPP2R1A	PPP2R2A
PRKD2	PRND	PRPSAP1	PSMB7	PSMC5	PSMD1	PSMD10	PSMD8	PTTG1IP	PWP1
RAB35	RALA	RALB	RALY	RAMP3	RANGAP1	RASIP1	RCN2	RGS11	RHOA
RHOC	RNF25	RNF34	RNPS1	ROBO4	RPL4	RPN2	RRAS2	SAE1	SAMD14
SCARF1	SEC61A1	SELE	SEMA6B	SEMA6C	SENP5	SH3GL1	SIN3B	SLC24A1	SLC25A6
SLC6A7	SMARCD1	SMARCE1	SNAPC4	SNTB2	SNTG2	SOX18	SPATS2	SPTBN5	SSBP1
STAB1	STK25	STRAP	STX12	SUMO3	SUN1	TACO1	TAF12	TAL1	TAOK2
TARBP2	TBC1D10B	TBX1	TDRD7	TEK	TFEC	THAP4	TIAL1	TIE1	TIPRL
TJP1	TMED9	TMEM109	TMEM115	TMEM39B	TNFSF18	TOR1AIP2	TPM3	TRAPPC3	TRPC4AP
TSPAN13	TSPO	TTLL5	TUSC2	TUT1	TXNDC9	TXNL1	UBAP2	UBE2E1	UBIAD1
UBXN1	UFD1L	UNC45A	URM1	USP5	VAMP3	VWF	WDR13	YIF1B	YKT6
YWHAE	ZC3H7B	ZDHHC24	ZFPL1	ZNF205	ZNF22	ZNF282	ZWILCH.		

Table S2. Clinical characteristics of high and low mvE cells CRC tumors in TCGA cohort.

TCGA	*Characteristics*	Low mvE cells (*n* = 446)	High mvE cells (*n* = 149)	*p*-value
**Age**	Median	68	66	0.409
	IQR	58-77	57-75	
**Race**	CA	219	64	0.157
	Black or African American	52	12	
	Asian	12	0	
	Unknown	163	73	
**Site**	Right	197	58	0.442
	Left	124	48	
	Rectum	110	40	
	Unknown	15	3	
**Histological type**	Adenocarcinoma	391	120	**0.021**
	Mucinous adenocarcinoma	47	27	
	Unknown	8	2	
**Genomic status**	MSS	299	105	0.533
	MSI	135	41	
	Unknown	12	3	
**AJCC T-category**	T1	18	2	0.061
	T2	78	24	
	T3	306	98	
	T4	43	25	
	Unknown	1	0	
**N-category**	N-	266	77	0.103
	N+	180	72	
	Unknown	0	0	
**M-category**	M-	331	112	0.74
	M+	111	34	
	Unknown	4	3	
**Stage**	I	80	23	0.396
	II	172	49	
	III	122	48	
	IV	59	24	
	Unknown	13	5	

AJCC, The American Joint Committee on Cancer; MSI, microsatellite instability; MSS, microsatellite stable; IQR, interquartile range.

Table S3. Clinical characteristics of high and low mvE cells CRC tumors in GSE39582 cohort.

**GSE39582**		**Low mvE cells**	**High mvE cells**	
**Characteristics**		(*n* = 424)	(*n* = 142)	*p*-value
**Age**	Median	69	68	0.555
	IQR	59-77	59-75	
**Genomic status**	dMMR	53	22	0.17
	pMMR	340	104	
	Unknown	31	16	
**AJCC**				

**T-category**	T1	9	2	0.151
	T2	40	5	
	T3	272	95	
	T4	92	27	
	Unknown	11	13	
**N-category**	N-	235	67	0.418
	N+	182	62	
	Unknown	7	13	
**M-category**	M-	367	115	0.524
	M+	49	12	
	Unknown	8	15	
**Stage**	0	4	0	0.209
	I	28	5	
	II	198	66	
	III	145	60	
	IV	49	11	
	Unknown	0	0	

AJCC, The American Joint Committee on Cancer; dMMR, deficient MMR (MSI-high tumors); MMR, mismatch repair; pMMR, proficient MMR (MSI-low and MSS tumors); IQR, interquartile range.

Table S4. Clinical characteristics of high and low mvE cells CRC tumors in GSE28072 cohort.

**GSE28072**		**Primary**			**Metastasis**		
		Low mvE cells	High mvE cells		Low mvE cells	High mvE cells	
Characteristics		(*n* = 42)	(*n* = 14)	*p*-value	(*n* = 20)	(*n* = 7)	*p*-value
Gender	Male	26	10	0.749	13	5	1.00
	Female	16	4		7	2	
metasttic site	Liver	-	-	-	18	5	0.383
	Lung	-	-	-	1	0	
	Peritoneum	-	-	-	1	2	

Table 5. Uni- and multivariate analysis in the TCGA cohort.

**TCGA, DSS**		**Univariate**				Multivariate			
**Factors**		**HR**	**95%CI**	*p*		HR	95%CI	*p*	
Age (y.o)	≧65 vs. < 65	1.56	0.96-2.51	0.071					
Subtype	Mucinous vs. Adeno	1.20	0.62-2.34	0.589					
Genomic status	MSI vs. MSS	1.23	0.76-1.99	0.39					
AJCC-Stage	III/IV vs. I/II	5.90	3.28-10.61	<0.001	*	5.71	3.17-10.27	<0.001	*
mvE cells	High vs. Low	2.14	1.34-3.42	0.001	*	1.84	1.14-2.98	0.001	*

*AJCC, The American Joint Committee on Cancer; CI, confidence interval; DSS, disease-specific survival; MSI, microsatellite instability; MSS, microsatellite stable; HR, hazard ratio; TCGA, The Cancer Genome atlas.

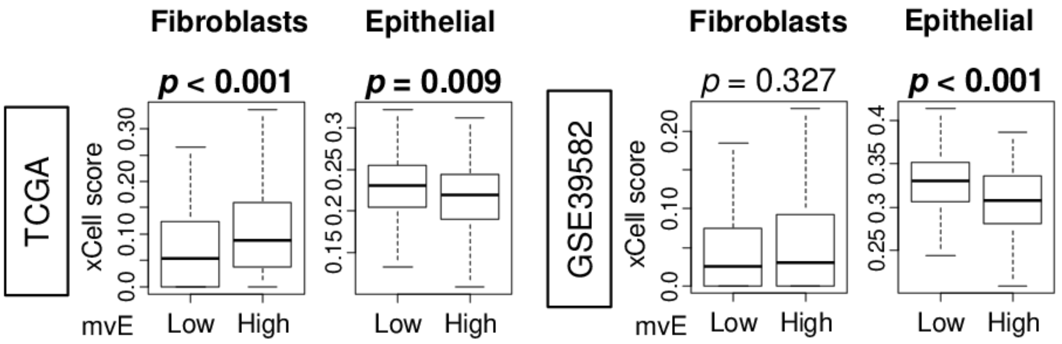

Figure S1. Association of the amount of mvE cells with fraction of fibroblasts and epithelial cells in the TCGA and GSE39582 cohorts. Boxplots of the fibroblasts and epithelial cells score by high and low mvE cell tumor groups. *P* value was calculated by Mann-Whitney U test.

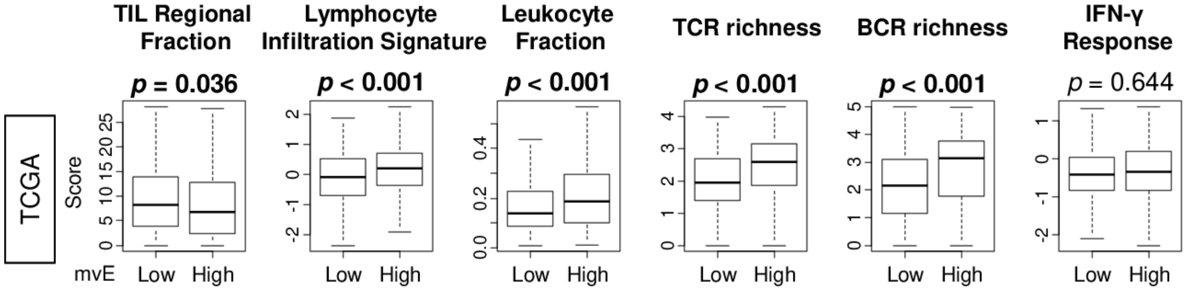

Figure S2. Association of the amount of mvE cells with immune-related score in the TCGA cohort. Boxplots of immune-related score; tumor infiltrating lymphocyte (TIL) regional fraction, lymphocyte infiltration, leukocyte fraction, T cell receptor (TCR) and B cell receptor (BCR) richness, and interferon (IFN)-γ response, by high and low mvE cell tumor groups in the TCGA cohort. *P* value was calculated by Mann-Whitney U test.

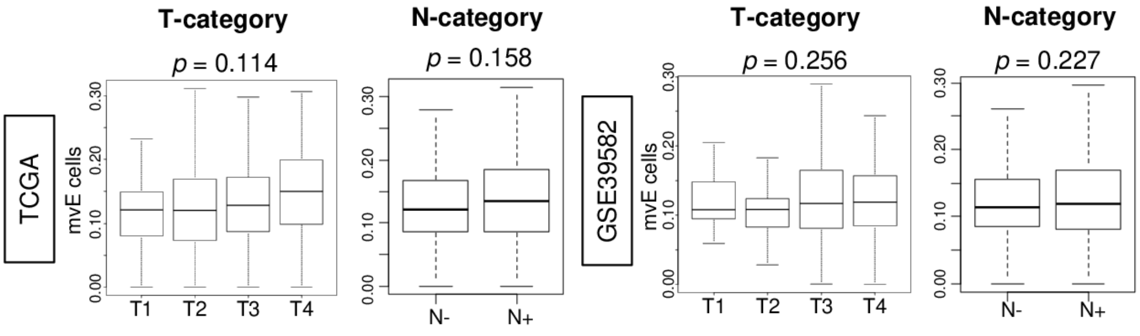

Figure S3. Association of the mvE cells with clinical aggressiveness in the TCGA and GSE39582 cohorts. Boxplots of the mvE cells by American Joint Committee on Cancer (AJCC) tumor size (T-category), and lymph node metastasis (N-category). *P*-value was calculated by Kruskal-Wallis test. .

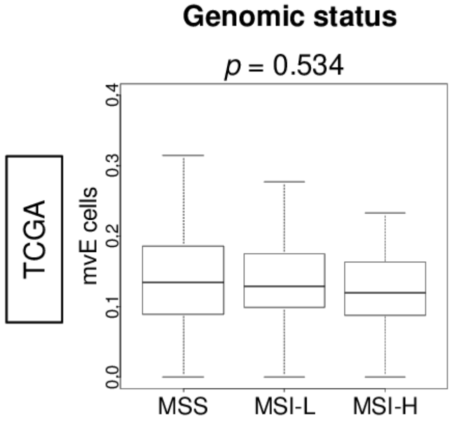

Figure S4. Association of the mvE cells with genomic status in the TCGA cohort**.** Boxplots of the mvE cells by microsatellite stable (MSS), microsatellite instability (MSI)-low (MSI-L), MSI-high (MSI-H). P-value was calculated by Kruskal-Wallis test. .

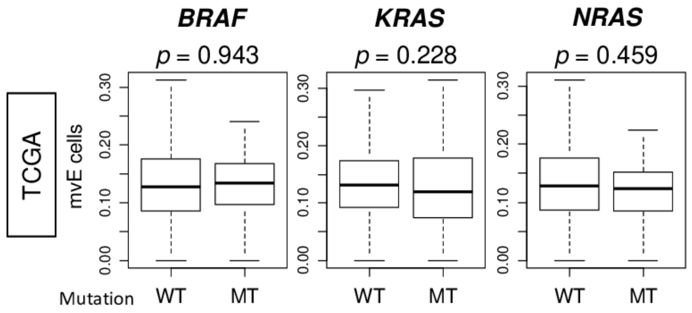

Figure S5. Association of the mvE cells with mutation of *BRAF, KRAS,* and *NRAS* in the TCGA cohort. Boxplots of mvE cells by non-mutation (WT) and mutation (MT) of *BRAF, KRAS,* and *NRAS* genes. *P* value was calculated by Mann-Whitney U test.

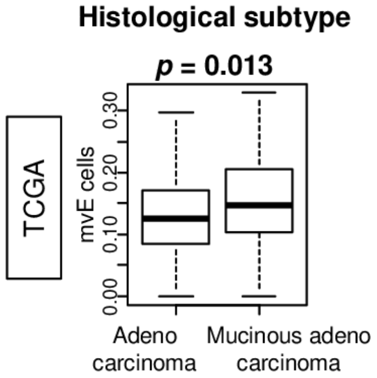

Figure S6. Association of the mvE cells with histological subtype in the TCGA cohort. Boxplots of the mvE cells by adenocarcinoma and mucinous adenocarcinoma. P value was calculated by Mann-Whitney U test.

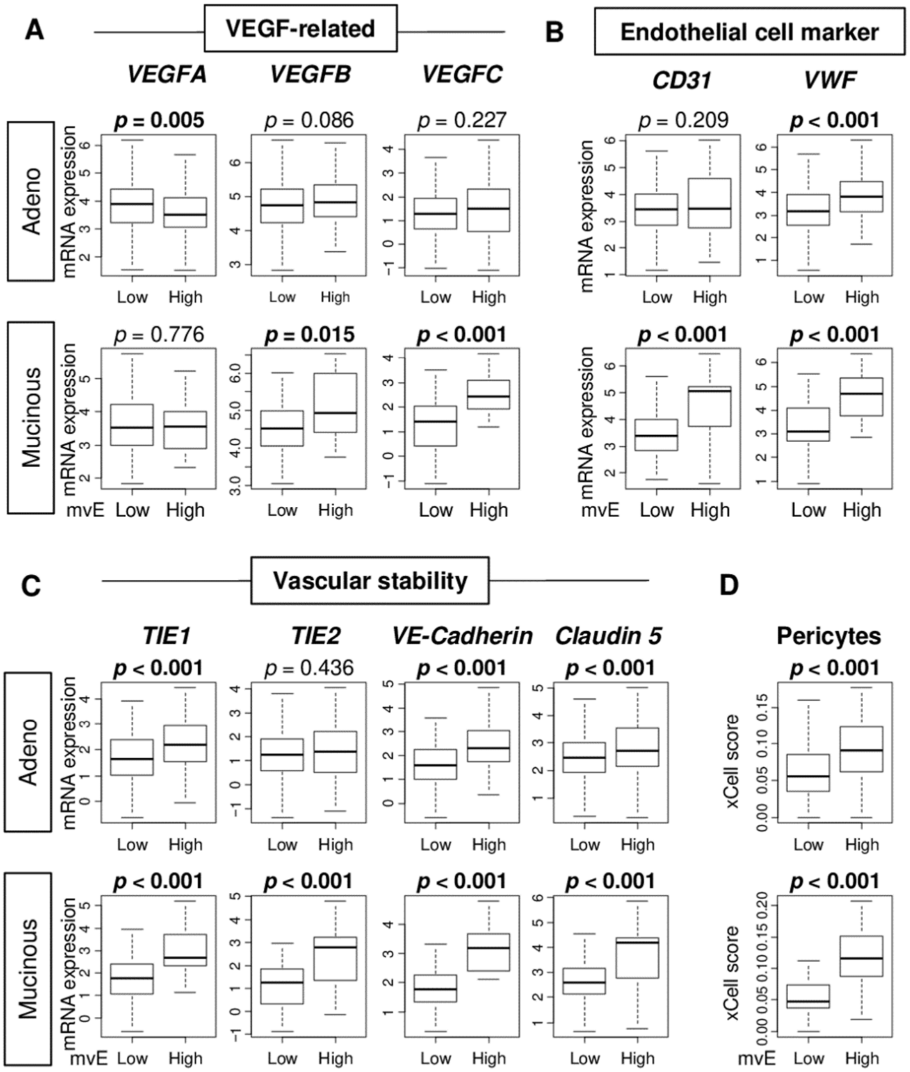

Figure S7. Association of the mvE cells with the expression of vessel-related genes in adenocarcinoma and mucinous adenocarcinoma. Boxplots of the comparison of **(A)** vascular endothelial growth factor (VEGFA)-related genes; *VEGFA, VEGFB, and VEGFC,* and **(B)** endothelial cell-related genes; *CD31* and *VWF*, and **(C)** vascular stability-related genes; *TIE1, TIE2*, VE-Cadherin, and Claudin 5, and **(D)** abundance of pericytes between high and low mvE groups in adenocarcinoma and mucinous adenocarcinoma. The top one-fourth was used as a cut-off to divide low and high groups for each cohort. *P* value was calculated by the Mann-Whitney U test.

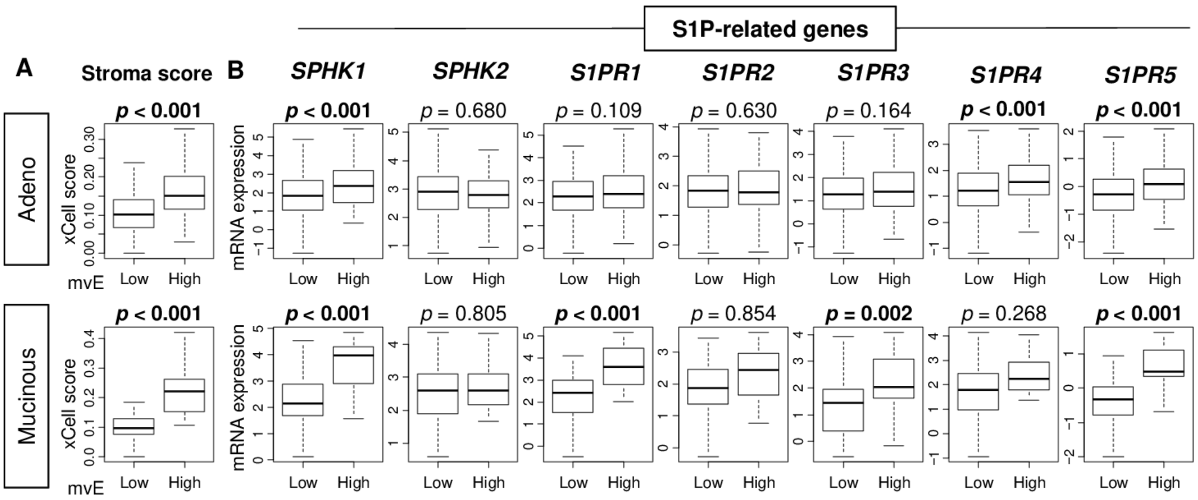

Figure S8. The association of the number of mvE cells with stromal cells and sphingosine-1-phosphate (S1P)-related genes in adenocarcinoma and mucinous adenocarcinoma. Boxplots of **(A)** the stromal score and **(B)** expression of S1P-related genes; *SPHK1, SPHK2, S1PR1, S1PR2, S1PR3, S1PR4, and S1PR5*, by high and low mvE cell groups in adenocarcinoma and mucinous adenocarcinoma. The top one-fourth was used as a cut-off to divide low and high groups for each cohort. *P*-value was calculated by the Mann-Whitney U test. S1PR, Sphingosine-1-phosphate kinase receptor.

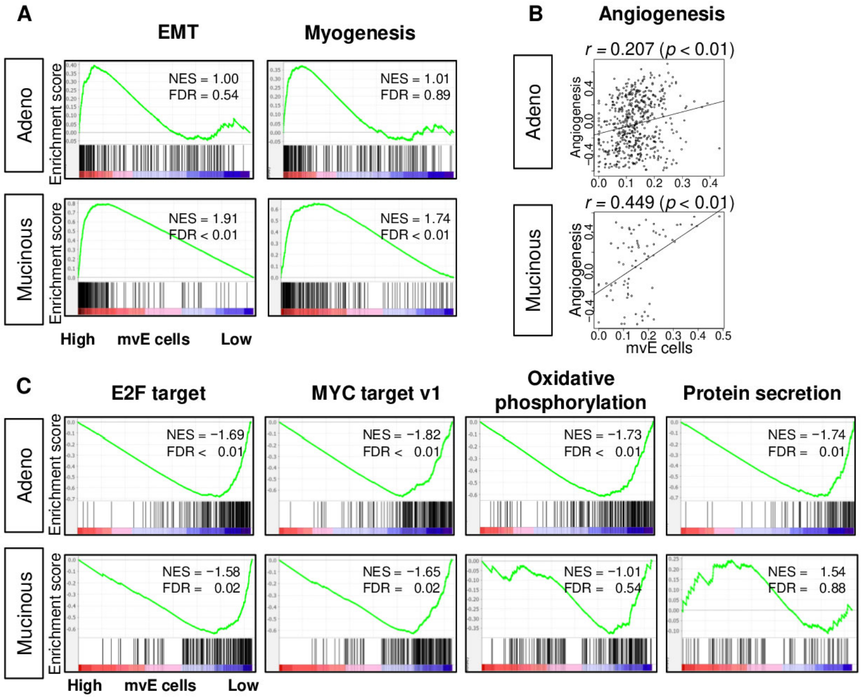

Figure S9. Gene set enrichment analysis (GSEA) of mvE cell CRC in adenocarcinoma and mucinous adenocarcinoma. (A) Enrichment plots of gene sets enriched in high mvE cell CRC and **(B)** Correlation plots between mvE cell score and angiogenesis score in

adenocarcinoma and mucinous adenocarcinoma. **(C)** Enrichment plots of gene sets enriched in low mvE cell CRC in adenocarcinoma and mucinous adenocarcinoma. Top one-fourth was used as a cut-off to divide low and high groups for each cohort in Figure A and C. Significantly enriched gene sets were selected based on false discovery rate (FDR) q-value < 0.25. Spearman's rank correlation test was used to analysis in figure B. EMT; epithelial-mesenchymal transition, NES; normalized enrichment score.
